# The Cancer Menace and What to Do*Read before the Wayne County Medical Society, April 1, 1918.

**Published:** 1918-09

**Authors:** J. H. Carstens

**Affiliations:** Detroit, Michigan. Professor of Abdominal and Pelvic Surgery, Detroit College of Medicine and Surgery


### The Cancer Menace and What To Do.*

J. H. CARSTENS, M. D., Detroit, Michigan.

Professor of Abdominal and Pelvic Surgery, Detroit College of Medicine and Surgery.

When we consider that one woman out of every eight dies of cancer, and one man out of every thirteen, the question looms up big to us, and it behooves us to do something to check this increase in cancer. In 1916 the death rate for cancer for every 100,000 population was eighty-one, making more than 80,000 for the whole country. Of course, tuberculosis has been decreased more than thirty per cent, but we know what causes tuberculosis, we do not know what causes cancer, and hence, we are very helpless. As Dr. Davis, Medical Director of the Amicable Life Insurance Company, Waco, Texas, says, "What are you doctors going to do about cancer? It is getting to be more and more prevalent, and is a disturbing problem for life insurance companies. Our losses are far greater than a generation ago. Can we keep any of those losses in our books by more thorough examinations?"

The Council of the Swiss Association for Combating Malignant Disease, asks for precise statistical and clinical information in regard to all fatal cases of cancer of the breast. Special notes are requested first of the influence of lactation, mastitis, or previous innocent tumors (fibroadenoma, cyst); on the developing of cancer growths; and, secondly, of the effect on prognosis of operation, roentgen rays or radium application.

Switzerland has, as well known, the highest cancer death rate in the world, although alone in the civilized countries, the rate is a diminishing one.

("Campaign notes of the Amer. Society for the Control of Cancer.")

Like all things, a cancer must have a beginning, one that is minute and cannot be seen. A cancerous growth gets to be the size of a millet seed, then that of a bean, then of a hickory nut, and thus continues to grow. When it is small no lymphatics are involved, and if we find it thus early and cut it out, the system is free of it, and continues so. Hence, at the present time the whole question of treatment resolves itself into that of early diagnosis; and the question of early diagnosis almost entirely depends upon the medical prac-

---

*Read before the Wayne County Medical Society, April 1, 1918.

titioner's **looking for the disease constantly.** I know that many people do not consult a physician, for they have no pain, and the disease is all too often far advanced before the doctor is consulted for these cases, and the profession cannot be blamed for those.

**Every medical man must constantly have before his mind the possibility of cancer,** and when that patient complains of soreness in the breasts, excessive flowing, or chronic irritation of the skin, **always think of cancer, cancer, cancer.** The greatest difficulty will be to educate the laity, and still, as it has been educated on the tuberculosis question, there should be no trouble to do the same thing with the cancer question, if only vigorous efforts are made.

I have advocated the creation of a certain day in the year to be called "Cancer Day," when every newspaper in the land should print an article on the subject, and every minister should preach from the pulpit on the cancer question, and every doctor should talk all that day wherever he goes on **cancer, cancer, and nothing but cancer.**

If every physician would thoroughly examine every case, he will be able to make a diagnosis early, when something can be done. If every woman with an excessive or an unusual vaginal discharge will be examined, if a little tissue be removed from the uterus which looks suspicious, and this be subjected to microscopic examination, many cases of cancer will be caught at the very beginning. And if every lump in the breast will be removed, every cyst, hardening or induration, or ulcer extirpated and subjected to a thorough pathologic examination, cancer will often thus be diagnosticated early. If every stomach or bowel trouble, instead of being allowed to drag along, would be subjected to a thorough investigation, if necessary even to an exploratory celiotomy, many more cancer cases would be recognized early.

When I say exploratory **celiotomy,** I do not mean that vicious exploratory operation that was recommended some years ago, of cutting into a tumor and removing a section, and then deciding whether it was malignant or not. This has been found the most vicious practice, as we get cancer implantation, and a rapid progress and spread of the disease. No tumor should be explored, it **should be removed with a large margin of tissue around it.** Great effort should be made **not to cut into the tumor,** or into any glands. After removal, an examination can be made microscopically, and the diagnosis made. This is especially applicable to tumors in the breast. Tumor in the breast requires complete removal of the breast with the glands; no chances should be taken. The only exceptions are those clear simple cases of fibroids found in young girls and young women, which can be removed under local anesthesia as a rule. In this connection, I might say that I am very much disgusted when I see a curetting done, or a lacerated cervix repaired, and the removed tissues thrown into a pail and not examined microscopically. I consider this **a most vicious practice.** I have repeatedly called attention to this fact in the past years, **that every curetting or tissue removed** from the cervix must be subjected to a microscopical examination, and cancer looked for. Ten years ago, I induced the State Board of Health to publish a cancer circular, after a great deal of trouble. This circular is still available, and should be made use of by the profession to distribute among the laity. **If we get the lay people to understand that an excessive flow, or irritating discharge, and a little enlargement or growth, or a continuous pain may mean cancer,** and that the medical man should be consulted early, much untold misery may be prevented and many lives prolonged. **It is carelessness that prevents the early recognition of this great scourge.**

Until we find the real cause or some specific cure, we are forced to preach the doctrine of early diagnosis and surgery, and in order to do that we must allow ourselves to be accused of having "cancer on the brain" by preaching, day in and day out, the story of cancer, cancer, cancer.

1447 David Whitney Building.

---

**Intestinal Parasitism in Troops in Service.** Vezeau of Lavergne, Le Prog. Med., Mch. 2, reports in 200 faecal examinations: no ova of ankylostomes; among 100 soldiers in the trenches, 73 carriers of trichocephalus, 7 of trichocephalus and ascaris, 8 of ascaris. Among men living in the sectors behind the trenches, in cantonments, 63% had trichocephalus, none ascaris. In the infected persons who showed no sign of disease, the trichocephalus ova varied from 1 or 2 to 5 in a preparation. Eosinophilia was never encountered in trichocephalus carriers, however great the number of parasites it was found in 4-9% of the carriers of ascaris. In 10 cases of diarrhoea without blood or fever, neither bacilli nor amoebae of dysentery were found but ova were numerous. In 8 of these cases, trichocephalus ova were numerous, 15-25 in a preparation. In 2 labeled "rebellious diarrhoea, colics" numerous ascaris ova were found. Thymol, calomel and santonin produced marked relief. In 14 cases of "febrile curve" ("fever undetermined in the U. S. military nomenclature") trichocephalus ova were found. Thymol relieved these.

